# A Molecular Basis for the Inhibition of Transient Receptor Potential Vanilloid Type 1 by Gomisin A

**DOI:** 10.1155/2017/6451905

**Published:** 2017-10-26

**Authors:** Sung Bae Lee, Shinhwa Noh, Hye Duck Yeom, Heejin Jo, Sanung Eom, Yoon Suh Kim, Sangsoo Nam, Hyunsu Bae, Jun-Ho Lee

**Affiliations:** ^1^Department of Biotechnology, Chonnam National University, Gwangju 61886, Republic of Korea; ^2^College of Korean Medicine, Kyung Hee University, Seoul 02447, Republic of Korea

## Abstract

Transient receptor potential (TRP) channel has critical actions as conditional sensors in primary afferent neurons. We studied the regulatory action of gomisin A on TRPV1 channel current in this report.* Schisandra chinensis* contains bioactive compounds such as the gomisin derivatives and their related compounds. Coapplication with gomisin A inhibited the capsaicin-mediated inward peak current. This inhibitory effect of gomisin A on capsaicin-induced inward current showed concentration-dependence and was reversible. The half maximal inhibitory concentration of gomisin A was 62.7 ± 8.4 *µ*M. In addition, this inhibition occurred in a noncompetition regulation mode and voltage insensitive manner. Furthermore, molecular docking studies of gomisin A on TRPV1 showed that it interacted predominantly with residues at cavities in the segments 1 and 2 of each subunit. Four potential binding sites for this ligand in the extracellular region at sensor domain of TRPV1 channel were identified. Point mutagenesis studies were undertaken, and gomisin A potency decreased for both the Y453A and N467A mutants. The double mutation of Y453 and N467 significantly attenuated inhibitory effects by gomisin A. In summary, this study revealed the molecular basis for the interaction between TRPV1 and gomisin A and provides a novel potent interaction ligand.

## 1. Introduction

TRPV1 receptor is the transient channel receptor subfamily 5 and it is reported as the receptor of capsaicin and vanilloid; nonselective cation channels became active by a wide diversity of exogeneity and endogeneity for physicochemical stimuli [1]. The representative regulators of TRPV1 are temperature, pH, and capsaicin, and its activation produced a painful and burning sensation. This receptor is responsible for the transmission or regulation of pain as well as the recognition of a variety of pain stimuli [2]. Various chemicals have a pharmacological effect by targeting this receptor, which is due to architectural resemblance with other ligand gate ion channel. This receptor has a three-dimensional structure that resembles voltage dependent ion channel very much because the membrane protein fragments 5 and 6 form ionic channels and act as pore region [3]. These center pores are enclosed by four different continuous domains, which act as voltage detectors during channel opening [4]. The common structural rearrangement of TRPs or voltage-gated ion channels are unknown, despite these structural similarities.

*Schisandra chinensis* is a traditional herbal medicine and spread out all around the world and cultivated in Far Eastern countries [5]. Recent studies have shown administration of* Schisandra chinensis* to have numerous beneficial medical effects, for example, as a neuroprotectant, on the significant heath progress of immune system and cardiovascular system. The lignans, schisandrin, and gomisins were chemical constituents of* Schisandra chinensis* [6]. In traditional medicine, the fruit of* Schisandra chinensis* is believed to treat diarrhea and lack of energy, arrests excessive sweating, refreshes the heart and kidneys, creates fluids, and reduces thirst [5].* Schisandra* elevated physical durability and provides stress protection for a wide range of injurious factors including thermal shock, scalding, frostbite, and immobilization [5, 7].* Schisandra chinensis* affects the immune system as well as nervous and gastrointestinal system [8–12].* Schisandra chinensis* contains bioactive compounds such as the gomisin derivatives and their related compounds. Recent studies reported many other lignans from the* Schisandra chinensis* and they showed the various biological regulatory effects.* Schisandra chinensis* is generally treated for a folk remedy use, but its concrete mechanisms in the physicochemical and pharmacological effects are not known in cell level.

The regulatory action of gomisin A on the TRPV1 receptor channel was examined in this study. Although* Schisandra chinensis* is used in folk remedy, it is not known if the folk usage is engaged in regulatory effects of ligand gated ion channels. Accordingly, TRPV1 receptor channel cRNAs were injected into* Xenopus* oocytes, and the regulatory action of gomisin A on the capsaicin-induced currents was examined. It was found that cotreatment of gomisin A attenuates the capsaicin-elicited, inward peak currents in reversible, voltage insensitive, concentration dependent, and noncompetitive manner.

## 2. Materials and Methods

### 2.1. Materials

Gomisin A was purchased from Wuhan ChemFac Biochemical (Hubei, China). Stock solutions of gomisin A of 100 mM concentration were prepared using DMSO. The plasmid DNA encoding the human TrpV1 receptor was obtained from OriGene. Other reagents were treated from Sigma-Aldrich or used as supplied, unless otherwise stated.

### 2.2. Preparation of* X. laevis* Oocytes and Mutagenesis of TRPV1 Receptors

The treatment of oocytes and mutagenesis were described in previous study [13]. Briefly, frogs caring procedures were followed by the Jonnam National University animal caring institute guidelines (JNU IACUC-YB-2017-07, July 2017). The removed oocyte from* X. laevis* was collagenized with rocking for 2 hrs in bath solution. The matured oocytes were selected and incubated in ND96 buffer with antibiotics. The introduction of complementary RNAs into the vegetable or animal pole of single cells was carried out using a microinjector. Oocyte recording experiments were tried out after 48 hrs for each expressed oocyte. The TRPV1 mutants were made by MAX-QuikChange mutagenesis protocol with Pfu DNA polymerase and desired mutation primers.

### 2.3. Molecular Docking Studies

The docking studies were investigated on Intel i7, PC with 16 GB RAM running the Windows 8 operating system, using AutoDock Tools (version 1.5) by the Scripps Institute. The protein structure of TRPV1 receptors was obtained from the Protein Data Bank (ID code 3J5P), and the 3D structure of the ligand (gomisin A) was obtained from Pubchem. The protein-ligand complex was programmed using AutoDock Tools and considered with minimized binding energy, inhibition constant, and intermolecular energy. The complex was analyzed using Ligplot (ver. 4.5.3) by EMBL-EBI and Pymol (ver. 1.8.4.2) by Schrödinger. Ligplot showed interactions between the protein and the ligand. Pymol was used to measure the distance between the complex and mutagenesis of amino acids of TRPV1 receptors.

### 2.4. Data Recording

The single cells were put in flowing buffer bath (The Warner Instruments) and flowed by ND96 medium at 1 ml/min. Each single oocyte was then penetrated with two thin electrodes filled with electrolyte solution. The microelectrodes resistance was from 0.5 to 0.8 MΩ. The electrophysiological experiment was performed at RT with Oocyte Clamp Amplifier and data collection was performed using Digidata 1320 and pClamp 9 (Molecular Devices, CA). For this study, the holding potential was clamped on −80 mV in each cell. The ramp experiments of the currents voltages relationship were shed from −80 to +60 mV of experiments of TRPV1 receptors current. Stock solution of 250 mM of gomisin A and used chemical compounds were prepared with DMSO and then they were diluted to each low concentration for actual use with ND96 bath buffer.

### 2.5. Data Analysis

The data analysis was described in previous study [13]. To acquire dose dependent curves for the effects of gomisin A on capsaicin-induced current, the induced peak currents at each gomisin A concentration were plotted using the Hill equation. A significance among the mean of the application values was measured using two-way ANOVA with Tukey tests of Origin pro 7.0 statistic software.

## 3. Results

### 3.1. Effect of Gomisin A on Control Oocytes and Oocytes Expressing Human TRPV1

**Figure 1:**
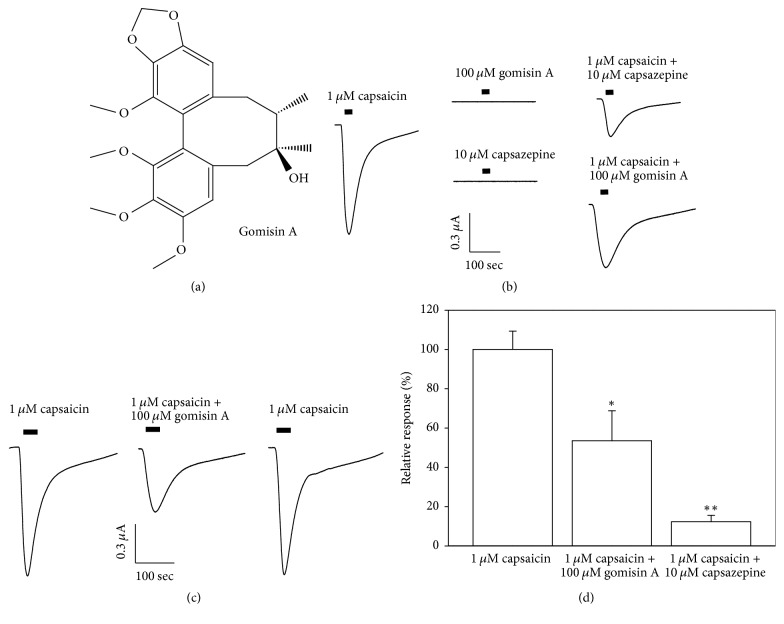
Effect of gomisin A on control oocytes or oocytes expressing human TRPV1 receptor channel. (a) Chemical structure of gomisin A. (b) Application of capsaicin (1 *μ*M) stimulated an inward current, whereas gomisin A (100 *μ*M) alone had no effect on oocytes expressing human TRPV1 receptor channel at a holding potential of −80 mV. Cotreatment of capsaicin (1 *μ*M) and capsazepine (10 *μ*M), which the respective TRPV1 receptor channel antagonist that blocked capsaicin-stimulated inward current, indicated that these receptors were functionally expressed in the experiment. (c) The effect of gomisin A on the capsaicin-stimulated inward current mediated by human TRPV1 receptor channel expressed in* Xenopus* oocytes. The coapplication of the oocytes with gomisin A (100 *μ*M) with 1 *μ*M capsaicin inhibited the capsaicin-induced inward current in a reversible manner (*n* = 9–12 from four different frogs). (d) The percent inhibition was 46.5 ± 4.5% and 79.5 ± 4.5% by 100 *μ*M gomisin A and 10 *μ*M capsazepine. The preapplication of gomisin A was 48.2 ± 7.2%, indication that either coapplication or preapplication causes the same extent of inhibition (data not shown). ^*∗*^*p* < 0.05, ^*∗∗*^*p* < 0.005 compared with response of capsaicin.

Initially, the two microelectrode voltage clamping methods were adapted to study the regulatory action of gomisin A on the oocytes that had been injected with 40 nl H_2_O (the control). Injection of capsaicin (1 *μ*M) or gomisin A (10 *μ*M) into oocytes did not result in any inward peak current. Coapplication of 1 *μ*M capsaicin and 10 *μ*M gomisin A also did not result in an inward current (data not shown). Then, the regulatory action of gomisin A against inward current was mediated by human TRPV1 receptor channel at membrane voltage potential as –80 mV. The treatment of 1 *μ*M capsaicin stimulated an inward current, and gomisin A (100 *μ*M) had no effect on currents of human TRPV1 receptor. Cotreatment of capsaicin (1 *μ*M) and capsazepine (10 *μ*M), a TRPV1 receptor channel antagonist, blocked the capsaicin-stimulated inward current, showing that expressed TRPV1 receptors were functionally operated in this experiment (Figure 1(b)).

### 3.2. Effect of Gomisin A on Capsaicin-Elicited Inward Peak Currents in Oocytes Expressing Human TRPV1 Receptor Channel

Next, the regulatory action of gomisin A on capsaicin-induced inward current arbitrated by human TRPV1 receptor channel was evaluated in a reversible manner. Coapplication of the oocytes with gomisin A (100 *μ*M) with 1 *μ*M capsaicin blocked the capsaicin-mediated current (Figures 1(c) and 1(d); *n* = 9–10 from nine different frogs). The percent inhibitory effect by gomisin A was 46.5 ± 4.5%. The preapplication of gomisin A was 48.2 ± 7.2%, showing that either coapplication or preapplication resulted in the same degree of inhibition (data not shown).

### 3.3. Current–Voltage Relationship and Concentration Dependency of the Capsaicin-Stimulated Inward Current in Response on Gomisin A Induced Inhibitory Effects

**Figure 2:**
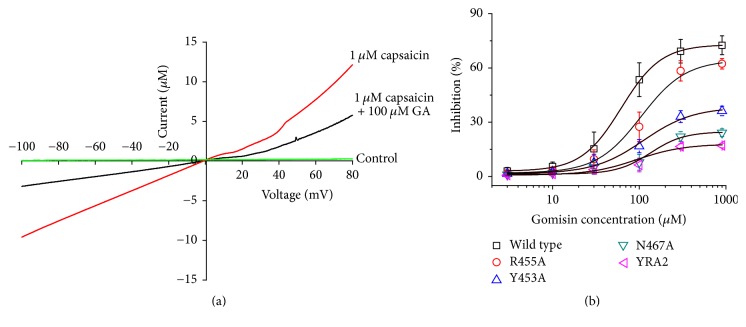
The voltage-dependency and concentration dependency of gomisin A on capsaicin-stimulated inward current in oocytes expressing human TRPV1 receptor channel. (a) The representative current–voltage relationship was obtained using 1 sec duration voltage ramps from −100 to +80 mV, at a holding potential of −80 mV. Voltage steps were applied before and after the application of capsaicin in the presence or absence of gomisin A (100 *μ*M). (b) The capsaicin-induced inward current in oocytes was elicited at a holding potential of −80 mV for the indicated time, in the presence of 1 *μ*M capsaicin, after which the indicated concentrations of gomisin A were coapplied with capsaicin. Concentration response curves for the effect of gomisin A on oocytes expressing the TRPV1 receptor. The percentage inhibition by gomisin A was calculated based on the average of the peak inward current elicited by capsaicin and that of the peak inward current elicited by capsaicin plus gomisin A. Each point represents the mean ± SEM (*n* = 7–12/group).

In further experimental process, we evaluate the mechanism by which gomisin A inhibited capsaicin-induced current of human TRPV1 receptor channel. The current–voltage relationship elicited by only 1 *μ*M capsaicin or by 1 *μ*M capsaicin plus gomisin A (100 *μ*M) was measured. These responses to treatment were then checked by holding membrane voltage potential as −80 mV and then increasing it from −100 to +80 mV on intensity of 1 sec (Figure 2(a)). The reversal membrane voltage potential of capsaicin-induced inward current was negative voltage near −10 mV. Coapplication of gomisin A with capsaicin blocked both inward (minus currents area) and outward (plus currents area) currents smaller than those induced by only capsaicin treatment. The inhibition of gomisin A on capsaicin-induced inward current was voltage insensitive. Gomisin A inhibited the capsaicin-induced inward current by 71.1 ± 16.2% at −100 mV and 42.6 ± 11.2% at + 80 mV (*n* = 7–9 from three different frogs). Concentration response tests elicited that coapplication with capsaicin plus different concentrations of gomisin A inhibited the capsaicin-stimulated inward peak current in human TRPV1 receptor (Figure 2(b)). The IC_50_ values were 62.1 ± 3.7 *μ*M for capsaicin-stimulated inward current. Hill coefficients were 1.9 ± 0.1 for the capsaicin-induced inward current (*n* = 8–12 from five different baths). It indicates that gomisin A inhibits the capsaicin-stimulated inward current in a voltage insensitive and concentration dependent way.

### 3.4. Dock Modeling of TRPV1 and Gomisin A

**Figure 3:**
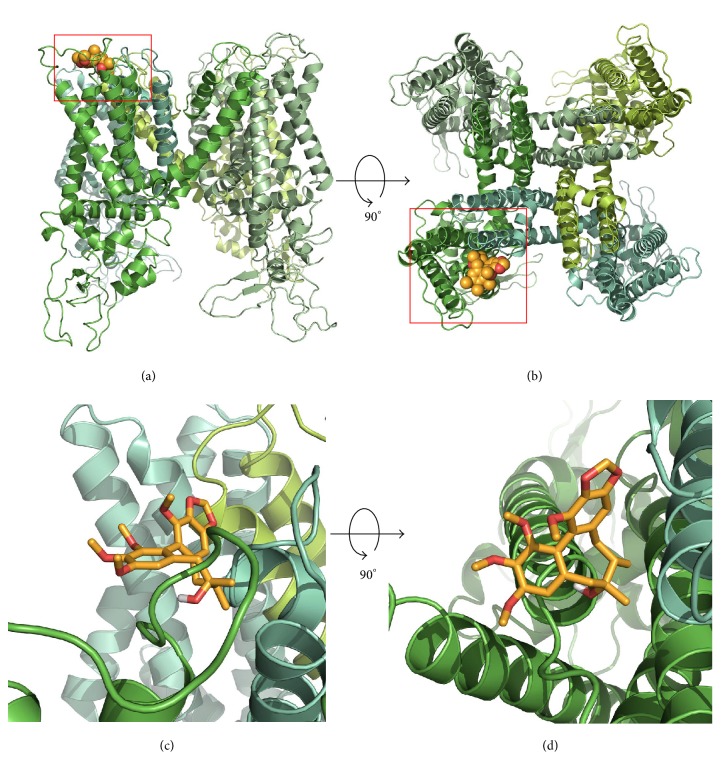
Computational molecular modeling of gomisin A docked to TRPV1 channel. (a and c) Side views of the docked gomisin A in complex with TRPV1 channel and (b and d) top views of docking model.

**Figure 4:**
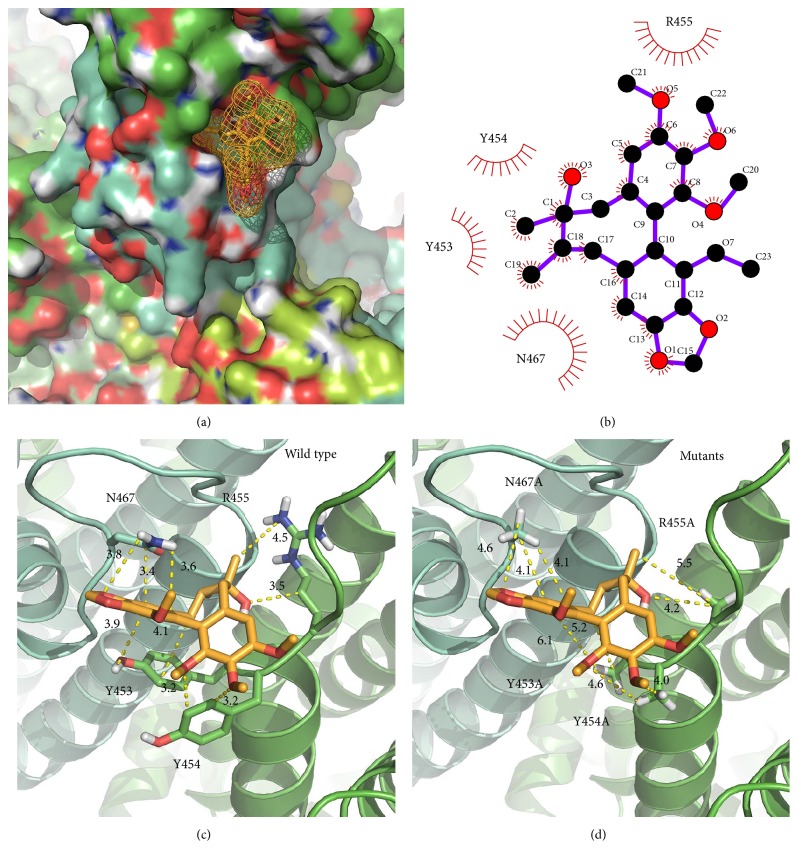
The binding pocket and docking results of gomisin A and TRPV1 channel. (a) Gomisin A located in binding pocket in extracellular area between segments 1 and 2 of TRPV1 channel. (b) 2D schematic presentation of the predicted binding mode of gomisin A in the ligand binding pocket. The ligands and important residues are shown. (c and d) Binding interface and gomisin A of the wild type (c) and the four mutant channels, whose mutations disturb the interaction with gomisin A to varying degrees.

To analyze the possible interaction way in gomisin A and TRPV1 channel, our team used wild type and mutant covalent docking homology modeling (Figure 3). The best-fit docking results showed gomisin A to form strong H bonds with wild type TRPV1, but not with mutants (Figure 4). The Y453, Y454, R455, and N467 residues in each subunit were assigned as the active site amino acid and it was set as 5 Å from those. Molecular docking revealed that gomisin A could fit into this pocket, interacting with previously unidentified residues, notably the positively charged amino acids from TRPV1 and hydroxyl group of gomisin A. In Figure 4(c), segment 1 or 2 of each TRPV1 subunits interacted with gomisin A, where each Y453 residue interacts with gomisin A (distance = 3.9 Å), Y454 (3.2 Å), R455 (3.5 Å), and N467 (3.4 Å).

### 3.5. Site-Directed Mutagenesis Study of TRPV1 and Gomisin A

To confirm the activity of each residue, the ability of gomisin A to regulate the current of a TRPV1 mutant in which each residue was replaced by an alanine residue was tested. The inhibitory effects of gomisin A on each of mutant channels is shown in Figure 2(b) and Table 1. The Y453A or N467A mutant showed significant attenuation of the inhibitory effects of gomisin A; the double mutation (YRA2) to alanine reduced the activity by 75.4%; the *V*_max_ value was reduced from 72.9 ± 1.0 to 17.9 ± 6.5; and the *n* value changed from 1.9 to 1.2. It indicates that gomisin A-mediated regulatory effects of TRPV1 channel activity were very closely associated with the Y453A or N467A residues.

## 4. Discussion

We demonstrated that gomisin A can regulate human TRPV1 receptor channel activity in* Xenopus* oocytes in the present report. Three pieces of hypothetical reasoning to support these conclusions were obtained. Firstly, gomisin A was found to impart a reversible, inhibitory effect (Figure 1) on TRPV1 activity. Secondly, gomisin A inhibited a capsaicin-induced inward current in TRPV1, in a concentration dependent way, and the inhibition of capsaicin-induced inward current by gomisin A occurred in a voltage insensitive way (Figure 2). Thirdly, in an in silico study, gomisin A interacted predominantly with residues at cavities in the segments 1 and 2 of each subunit of TRPV1 channel. This pocket indicated four potential binding sites, and mutants of Y453A or N467A showed significantly reduced activity of gomisin A on currents of TRPV1 channel.

*Schisandra chinensis* contained gomisin derivatives, and the berries were associated with the five flavors such as sweet, sour, bitter, astringent, and salty. Thus, the taste of acidic and salty were known hepatoprotective effects, while the bitter and puckery tastes have curative effects on the cardiovascular system, and the sweet flavor has a curative effect on the gastric system [5, 7]. Specifically,* Schisandra chinensis* has protective effects on cold stress-induced malondialdehyde in hepatic system [14, 15] and antioxidant activity [16], hepatic protective [5, 17–19], myocardial protective [17, 20], antitumor [21–23], antivirus [24], and anti-inflammatory [25] actions. In neuronal system, gomisin A was shown to reverse scopolamine-induced cognitive impairments in rodents [26, 27]. The beneficial facts of* Schisandra chinensis* suggest that gomisin derivatives can be treated on memory impairment and the protective effects as medications are intermediated by protective improvement of nerve system.

As mentioned previously, capsaicin evokes the sensation of burning pain and hyperalgesia when applied subcutaneously or intradermally [3, 28]. These effects are mediated predominantly by the action of capsaicin at primary nociceptive afferent neurons [29, 30]. The enhancement of capsaicin-stimulated currents in peripheral sensory-neurons usually results in the release of more pain-causing neurotransmitters. Previous reports showing the neuroprotective effect of gomisin A, and its capacity to attenuate neuronal related behavior induced by capsaicin treatment, are more likely to be due to their interaction at the sites of the central nervous system. Moreover,* Schisandra chinensis* can recover hypersensitive stomach ache induced by intestine 5-HT pathway in rats [31]. However, the present results do not inform how the* in vitro* improvement of capsaicin-stimulated currents by gomisin A relates to the* in vivo* inhibition of the capsaicin-stimulated, agony related behavior. One possibility is that gomisin A might interact with the TRPV1 receptor at the spinal level. Thus,* in vivo* administration of gomisin A at the central nervous system prior to administration of capsaicin might positively exert an effect on TRPV1 receptor channel activity.

Many previous reports show that various agents regulate ligand gated-ion channels expressed in* Xenopus* oocytes [32, 33]. These results indicate that gomisin A may play a role in regulation of neurotransmitter release or excitable neuron induced by TRPV1 receptor channel activation. In addition, our research results show that gomisin A can be used as a therapeutic medication for the relief TRPV1 receptor channel-related clinical symptoms.

In conclusion, we have shown that gomisin A inhibits capsaicin-induced inward current by interacting with segments 1 and 2 of the TRPV1 receptor channel. These inhibitory effects of gomisin A on the agonist gated-gated ion channel may explain some of its pharmacological effects.

## Figures and Tables

**Table 1:** Effects of gomisin A on mutant TRPV1 receptor channel.

	*V*max	IC_50_	*n*
Wild type	72.9 ± 1.0	62.1 ± 3.7	1.9 ± 0.1
A452Y	79.2 ± 3.1	94.7 ± 12.3	2.2 ± 0.8
Y453A	38.3 ± 2.4	111.6 ± 19.5	1.2 ± 0.5
Y454A	59.2 ± 3.0	94.7 ± 9.5	2.2 ± 0.8
R455A	64.4 ± 2.6	82.5 ± 8.2	1.7 ± 0.6
V456A	91.5 ± 2.6	72.2 ± 9.2	1.8 ± 0.8
L465A	68.3 ± 3.5	78.2 ± 4.6	1.8 ± 0.3
K466A	64.7 ± 2.1	81.5 ± 5.9	2.3 ± 0.5
N467A	24.7 ± 2.0	134.7 ± 12.5	1.3 ± 0.5
T468A	67.9 ± 1.5	94.3 ± 8.2	1.6 ± 0.5
V469A	72.5 ± 5.6	82.5 ± 12.5	2.1 ± 0.7
YNA2	17.9 ± 6.5	193.9 ± 33.5	1.2 ± 1.5

Values represent the means ± SEM (*n* = 7–12/group). Currents were elicited at a holding potential of −80 mV. IC_50_ (*μ*M), *V*_max_, and Hill coefficient values were determined as described in Materials and Methods.

## References

[1] Chávez A. E., Chiu C. Q., Castillo P. E. (2010). TRPV1 activation by endogenous anandamide triggers postsynaptic long-term depression in dentate gyrus. *Nature Neuroscience*.

[2] Park C.-K., Lü N., Xu Z.-Z., Liu T., Serhan C. N., Ji R.-R. (2011). Resolving TRPV1- and TNF-*α*-mediated spinal cord synaptic plasticity and inflammatory pain with neuroprotectin D1. *The Journal of Neuroscience*.

[3] Cao E., Liao M., Cheng Y., Julius D. (2013). TRPV1 structures in distinct conformations reveal activation mechanisms. *Nature*.

[4] Catterall W. A. (2010). Ion channel voltage sensors: Structure, function, and pathophysiology. *Neuron*.

[5] Panossian A., Wikman G. (2008). Pharmacology of *Schisandra chinensis* Bail.: an overview of Russian research and uses in medicine. *Journal of Ethnopharmacology*.

[6] Lu Y., Chen D.-F. (2009). Analysis of *Schisandra chinensis* and *Schisandra sphenanthera*. *Journal of Chromatography A*.

[7] Chun J. N., Cho M., So I., Jeon J.-H. (2014). The protective effects of Schisandra chinensis fruit extract and its lignans against cardiovascular disease: a review of the molecular mechanisms. *Fitoterapia*.

[8] Li X., Fan X., Li D. (2016). Schisandra sphenanthera extract facilitates liver regeneration after partial hepatectomy in mice. *Drug Metabolism and Disposition*.

[9] Kwan H. Y., Niu X., Dai W. (2015). Lipidomic-based investigation into the regulatory effect of Schisandrin B on palmitic acid level in non-alcoholic steatotic livers. *Scientific Reports*.

[10] Giridharan V. V., Thandavarayan R. A., Arumugam S. (2015). Schisandrin B ameliorates ICV-infused amyloid *β* induced oxidative stress and neuronal dysfunction through inhibiting RAGE/NF-*κ*B/MAPK and Up-regulating HSP/beclin expression. *PLoS ONE*.

[11] Zhao T., Feng Y., Li J. (2014). *Schisandra* polysaccharide evokes immunomodulatory activity through TLR 4-mediated activation of macrophages. *International Journal of Biological Macromolecules*.

[12] Zhang C., Mao X., Zhao X. (2014). Gomisin N isolated from Schisandra chinensis augments pentobarbital-induced sleep behaviors through the modification of the serotonergic and GABAergic system. *Fitoterapia*.

[13] Yeom H. D., Lee J.-H. (2016). Regulation of human Kv1.4 channel activity by the antidepressant metergoline. *Biological & Pharmaceutical Bulletin*.

[14] Lupandin A. V. (1991). The general mechanism of body adaptation under the influence of polyphenol adaptogens. *Uspekhi Fizicheskikh Nauk*.

[15] Lupandin A. V. (1990). The adaptation to extreme natural and technogenic factors in trained and untrained subjects under the influence of adaptogens. *Fiziologiia cheloveka*.

[16] Kim J.-H., Choi Y.-W., Park C. (2010). Apoptosis induction of human leukemia U937 cells by gomisin N, a dibenzocyclooctadiene lignan, isolated from Schizandra chinensis Baill. *Food and Chemical Toxicology*.

[17] Liu G.-T., Zhang T.-M., Wang B.-E., Wang Y.-W. (1992). Protective action of seven natural phenolic compounds against peroxidative damage to biomembranes. *Biochemical Pharmacology*.

[18] Kim M., Lim S. J., Lee H.-J., Kim S. Y., Nho C. W. (2015). Gomisin J Inhibits Oleic Acid-Induced Hepatic Lipogenesis by Activation of the AMPK-Dependent Pathway and Inhibition of the Hepatokine Fetuin-A in HepG2 Cells. *Journal of Agricultural and Food Chemistry*.

[19] Teng H., Lee W. Y. (2014). Antibacterial and antioxidant activities and chemical compositions of volatile oils extracted from Schisandra chinensis Baill. seeds using simultaneous distillation extraction method, and comparison with Soxhlet and microwave-assisted extraction. *Bioscience, Biotechnology, and Biochemistry*.

[20] McCord J. M. (1988). Free radicals and myocardial ischemia: overview and outlook. *Free Radical Biology & Medicine*.

[21] Yim N.-H., Kim A., Jung Y. P., Kim T., Ma C. J., Ma J. Y. (2015). Fermented So-Cheong-Ryong-Tang (FCY) induces apoptosis via the activation of caspases and the regulation of MAPK signaling pathways in cancer cells. *BMC Complementary and Alternative Medicine*.

[22] Liu X., Ni C., Li C., Liu T. (2015). Drug-drug interation prediction between ketoconazole and anti-liver cancer drug gomisin G. *African Health Sciences*.

[23] Casarin E., Dall'Acqua S., Šmejkal K., Šlapetová T., Innocenti G., Carrara M. (2014). Molecular mechanisms of antiproliferative effects induced by Schisandra-derived dibenzocyclooctadiene lignans (+)-deoxyschisandrin and (-)-gomisin N in human tumour cell lines. *Fitoterapia*.

[24] Bai R., Zhang X.-J., Li Y.-L. (2015). SJP-L-5, a novel small-molecule compound, inhibits HIV-1 infection by blocking viral DNA nuclear entry Microbe-host interactions and microbial pathogenicity. *BMC Microbiology*.

[25] Wang X., Hu D., Zhang L. (2014). Gomisin A inhibits lipopolysaccharide-induced inflammatory responses in N9 microglia via blocking the NF-*κ*B/MAPKs pathway. *Food and Chemical Toxicology*.

[26] Egashira N., Kurauchi K., Iwasaki K. (2008). Schizandrin reverses memory impairment in rats. *Phytotherapy Research*.

[27] Kim D. H., Hung T. M., Bae K. H. (2006). Gomisin A improves scopolamine-induced memory impairment in mice. *European Journal of Pharmacology*.

[28] Bevan S., Szolcsányi J. (1990). Sensory neuron-specific actions of capsaicin: mechanisms and applications. *Trends in Pharmacological Sciences*.

[29] Cao E., Cordero-Morales J. F., Liu B., Qin F., Julius D. (2013). TRPV1 channels are intrinsically heat sensitive and negatively regulated by phosphoinositide lipids. *Neuron*.

[30] McKemy D. D., Neuhausser W. M., Julius D. (2002). Identification of a cold receptor reveals a general role for TRP channels in thermosensation. *Nature*.

[31] Yang J.-M., Xian Y.-F., Ip P. S. P. (2012). Schisandra chinensis reverses visceral hypersensitivity in a neonatal-maternal separated rat model. *Phytomedicine*.

[32] Sobczak K., Bangel-Ruland N., Leier G., Weber W.-M. (2010). Endogenous transport systems in the Xenopus laevis oocyte plasma membrane. *Methods*.

[33] Weber W.-M. (1999). Ion currents of Xenopus laevis oocytes: State of the art. *Biochimica et Biophysica Acta (BBA) - Biomembranes*.

